# Progress in lead-free piezoelectric nanofiller materials and related composite nanogenerator devices

**DOI:** 10.1039/c9na00809h

**Published:** 2020-04-29

**Authors:** Yong Zhang, Hyunseung Kim, Qing Wang, Wook Jo, Angus I. Kingon, Seung-Hyun Kim, Chang Kyu Jeong

**Affiliations:** State Key Laboratory of Silicate Materials for Architectures, Center for Smart Materials and Device Integration, School of Materials Science and Engineering, Wuhan University of Technology Wuhan 430070 China; Department of Materials Science and Engineering, National University of Singapore 9 Engineering Drive 1 117575 Singapore; Hydrogen and Fuel Cell Research Center, Department of Energy Storage/Conversion Engineering, Jeonbuk National University Jeonju Jeonbuk 54896 Republic of Korea ckyu@jbnu.ac.kr; Department of Materials Science and Engineering, The Pennsylvania State University, University Park PA 16802 USA; School of Materials Science and Engineering, Jülich-UNIST Joint Leading Institute for Advanced Energy Research (JULIA), Ulsan National Institute of Science and Technology (UNIST) Ulsan 44919 Republic of Korea; School of Engineering, Brown University Providence RI 02912 USA seunghyun_kim@brown.edu; Division of Advanced Materials Engineering, Jeonbuk National University Jeonju Jeonbuk 54896 Republic of Korea ckyu@jbnu.ac.kr

## Abstract

Current piezoelectric device systems need a significant reduction in size and weight so that electronic modules of increasing capacity and functionality can be incorporated into a great range of applications, particularly in energy device platforms. The key question for most applications is whether they can compete in the race of down-scaling and an easy integration with highly adaptable properties into various system technologies such as nano-electro-mechanical systems (NEMS). Piezoelectric NEMS have potential to offer access to a parameter space for sensing, actuating, and powering, which is inflential and intriguing. Fortunately, recent advances in modelling, synthesis, and characterization techniques are spurring unprecedented developments in a new field of piezoelectric nano-materials and devices. While the need for looking more closely at the piezoelectric nano-materials is driven by the relentless drive of miniaturization, there is an additional motivation: the piezoelectric materials, which are showing the largest electromechanical responses, are currently toxic lead (Pb)-based perovskite materials (such as the ubiquitous Pb(Zr,Ti)O_3_, PZT). This is important, as there is strong legislative and moral push to remove toxic lead compounds from commercial products. By far, the lack of viable alternatives has led to continuing exemptions to allow their temporary use in piezoelectric applications. However, the present exemption will expire soon, and the concurrent improvement of lead-free piezoelectric materials has led to the possibility that no new exemption will be granted. In this paper, the universal approaches and recent progresses in the field of lead-free piezoelectric nano-materials, initially focusing on hybrid composite materials as well as individual nanoparticles, and related energy harvesting devices are systematically elaborated. The paper begins with a short introduction to the properties of interest in various piezoelectric nanomaterials and a brief description of the current state-of-the-art for lead-free piezoelectric nanostructured materials. We then describe several key methodologies for the synthesis of nanostructure materials including nanoparticles, followed by the discussion on the critical current and emerging applications in detail.

## Abstract 2

This report is a representative review article which deeply describes lead-free piezoelectric nanofillers and related composite nanogenerator devices.

## Graphical abstract

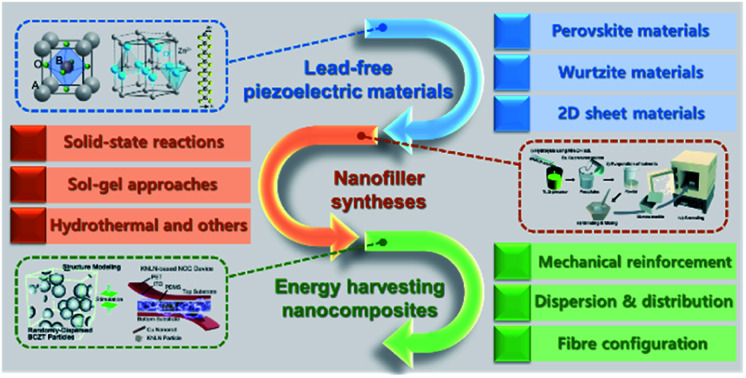

## 1. Introduction

The piezoelectric effect, which was first identified by Jacques and Pierre Curie in 1880, offers near instantaneous conversion of electromechanical energy with extreme precision and is thus utilized in many applications such as accelerometers, actuators, fuel injectors, motors, resonators, sensors, transducers and energy harvester, *etc.*^1–4^ In general, piezoelectric materials are simply divided into four categories: ceramics, polymers, single crystals, and organic–inorganic hybrid composites. Among them, piezoelectric ceramics have gained a huge popularity in the global market due to high piezoelectric performances and electromechanical conversion efficiencies, easy morphology- and property-tunability, low manufacturing cost, and easy mass production.^5,6^ So far, piezoelectrics have been traditionally dominated by Pb(Zr,Ti)O_3_ (PZT), which contains ∼60 wt% of environmentally hazardous lead.^7,8^ In several countries, including European Union (EU) and the U.S. state California, the directive Restriction on the Use of Certain Hazardous Substances in Electrical and Electronic Equipment (RoHS II) or an equivalent has been implemented in the legislation.^9^ According to the regulations, the concentration of lead in homogeneous material in the household or industrial devices must not exceed 0.1 wt%.^3^ Piezoelectrics for particular applications have been granted an exemption until a feasible alternative for lead-based materials are ready for use.^10,11^ This caused a huge burst in the research of lead-free piezoelectric materials in the last 30 years. Even if there are no viable lead-free piezoelectric material alternatives yet, recent encouraging results have demonstrated that some of the lead-free piezoelectric ceramic materials are beginning to approach PZT in performance.^12–15^ In addition to piezoelectric bulk ceramic materials, the usage of lead-based piezoelectric nanofillers including nanoparticles for hybrid composites has also suffered from the strict inspection due to toxicity of lead and is expected to have the same restriction in the near future, implying an urgent and emerging need for finding eco-friendly lead-free nanomaterial alternatives. Therefore, an insight-driven and massively parallel research trend of eco-friendly and size-scaled material systems has been expanded to nanoscience and nanotechnology. Specifically, perovskite-type piezoelectric nanostructured materials have attracted great interests due to their potential properties – including size, shape, optical and thermal responses, and dielectric and piezoelectric characteristics, which can be controlled by external forces such as electric field and physical stimulus representatively pressure, though not limited to pressure, strain/stress, vibration, and torsion *etc.* One of most important features is that these piezoelectric nanostructure materials can generate a factor of four to six increase in the extrinsic piezoelectric response compared to polycrystalline thin films.

Stretching our collective investigations to take a full advantage of nanoscale devices (*e.g.*, nano-electro-mechanical systems (NEMS), nanogenerators, *etc.*) demand the exploration of novel transduction mechanism, fundamental understanding of nanostructure materials, fabrication of nanoscale materials and devices, and future perspectives.^16–18^ Piezoelectric nanomaterials (PNs) are generally classified into several categories based on a diverse range of configurations and dimensions, *i.e.*, zero-dimensional (0D) PNs (nanoparticles and nanocubes), one-dimensional (1D) PNs (nanowires, nanofibers, nanorods and nanotubes) and two-dimensional (2D) PNs (nanoplates and nanofilms).^19^ Isotropic 0D nanoparticles with various sizes represent the most common nanoscale configuration for current massive applications and commercial products due to relatively simple synthesis flow and high-yield and high-quality manufacturing.^20^ These PNs with stringent process and quality controls have laid the foundation of a myriad of piezoelectric NEMS applications such as nanoactutors, nanosensors, nanoprobes, nanoresonators and nanogenerators, *etc.*^21^ In particular, nanogenerators, which were initially proposed by Z. L. Wang's research group in 2006, have become one of the hottest topics in materials and energy related research community.^22^ Piezoelectric nanogenerators are defined as mechanical energy harvesting devices with promising abilities for harvesting random mechanical energy into electric energy by utilizing piezoelectric effect or other electromechanical coupling phenomena.^23,24^ They have demonstrated high potentials for driving small-scale and low power electronics, wireless sensor networks, and implantable biomedical devices with self-powering capability.^25–31^ While a significant headway has been made in the field of nanogenerators, the amount of energy produced in most cases is still not sufficient to power the desired electronic systems. Therefore, a number of research strategies (through optimal selections of high-performance piezoelectric materials, ordered arrangement of materials, device structures, theoretical simulations, *etc.*) have been proposed and performed to improve the overall output power of piezoelectric energy harvesters, which will be summarized in the following sections.^32–34^

As described above, lead-free piezoelectric nanomaterials and related energy harvesting devices have attracted great attention with recent burgeoning advances.^35^ Although the vast majority of piezoelectric materials are mainly perovskite and wurtzite structures,^36–39^ the importance of piezoelectric phenomena has triggered a broad search for other non-traditional piezoelectric material systems such as 2D materials (*e.g.*, hexagonal boron nitrate (h-BN) and molybdenum disulfide (MoS_2_))^40,41^ and biomaterials (elastin, virus, spider silk, *etc.*)^42,43^ for unprecedented levels of new piezoelectric properties.^44–47^ It is important to note that the underlying physics in and related electromechanical coupling mechanisms for such new material systems, which are complex and not fully understood, need to be clarified further through more systematic investigations to enhance our understanding in this research field.^48^

Even though there have been a number of reviews on lead-free piezoelectric nanomaterials due to high potentials for future eco-friendly and biocompatible electronic applications,^11,49,50^ the understanding and the control of piezoelectric material properties at nanometer-scale are still at a very basic level, with the majority of reports just focusing on overcoming the processing difficulties. In addition, a fundamental understanding of the size effects on the functional piezoelectric response is still needed since limited knowledge exists on the origins of the performance of and the mechanisms for piezoelectric nanomaterials and devices due to the complex nature of materials and difficulty in fabrication at nanoscales. Moreover, the extensive research on this field during the last several years has intrigued new phenomena through recent advances in modelling, synthesis and characterization techniques, which have not been included in the previous reviews to date. Thus, this review article explores how the study of piezoelectric nanomaterials, specifically nanoparticles and nano-composites, has expanded our understanding of fundamental effects, enabled the discovery of novel phases and physics, and allowed unprecedented control of properties. This review also discusses several exciting possibilities for the development of new materials and devices, including those in transduction sensors and energy harvesting devices. This article provides a comprehensive overview and summary of the investigations on the universal approaches to and recent progresses of lead-free piezoelectric nanoparticles and related energy harvesting devices. We believe that this review can be an excellent research training vehicle, as it demands both research depth and breadth, and covers multidisciplinary topics ranging from fundamental studies of nanoscale piezoelectric materials, and process optimization to advanced application aspects such as device structures, fabrication methodologies, and design elements of energy harvesting devices (nanogenerators). It provides the researchers with knowledge and awareness of scale issues of the functional structure in current miniaturized electronic devices where the size is receiving increasing attention. It should be noted that various shapes of lead-free piezoelectric nanomaterials such as nanowires, nanorods and nanosheets are considered as nanoparticles in this review, because they can be regarded as 1D or 2D nanoparticles in general.^51^ Finally, this paper highlights future challenges and perspectives for successful implementation of piezoelectric nanomaterials in energy harvesting device applications in the last part. It is expected to yield key insights into the science of the phenomenon, including resolving the current issues regarding scaling effects and practical device applications. We hope that this comprehensive review would provide a timely update and a valuable guidance for the scientists and engineers who are working in the field of piezoelectric nanomaterials and related energy devices.

## 2. Lead-free piezoelectric nanomaterials

### 2.1 Types of lead-free piezoelectric nanomaterials

Lead-free piezoelectric nanomaterials could be simply divided into several groups according to their crystal configuration: perovskite structure, wurtzite structure, and new material systems. In this paper, the new systems (*i.e.* 2D materials) are typically defined as diverse compositions which cannot be affiliated with perovskite and wurtzite structures. We will summarize them one by one from the lesson of brief history to the currently studied level. Note that the piezoelectricity of bismuth-layered structures (*e.g.* Bi_4_Ti_3_O_12_) and tungsten bronze structures (*e.g.* KSr_2_Nb_5_O_15_) are not within the scope of this review because the two material families have been mainly identified in bulk materials.^52^

#### 2.1.1 Perovskite structure

**Fig. 1:**
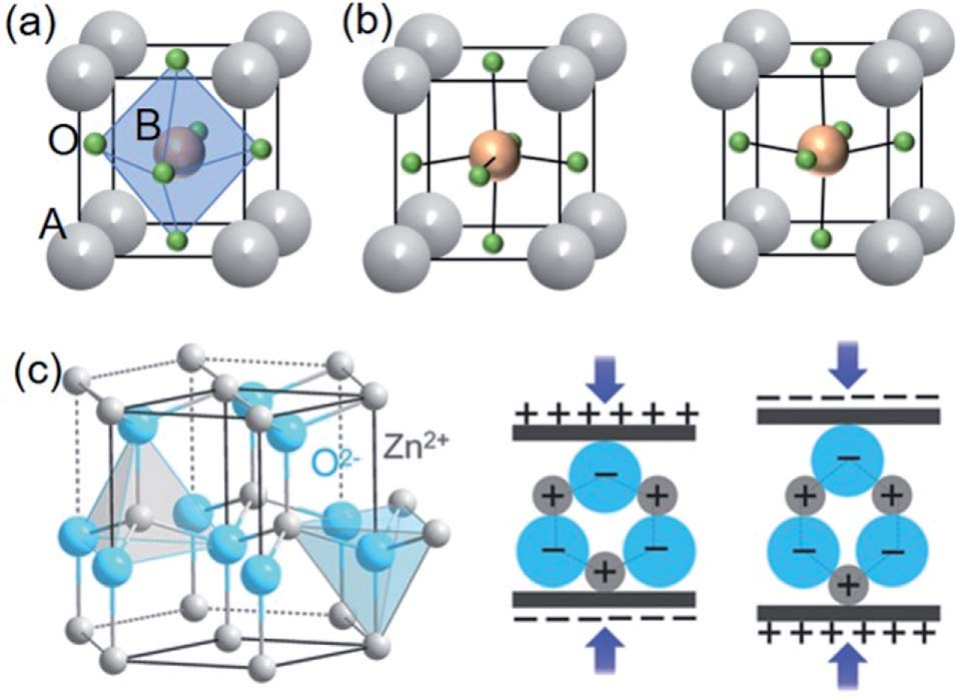
Stereoscopic illustration of ABO_3_ perovskite structures (a) with centrosymmetry and (b) with non-centrosymmetry by B ions upwards or downwards. (c) Schematics of wurtzite structure (ZnO as an example) and the related piezoelectric effect. This figure has been adapted/reproduced from ref. 53 with permission from John Wiley & Sons, copyright 2016.

Piezoelectric materials with the perovskite structure are most widely studied and technologically utilized for various applications nowadays. Fig. 1a shows the perovskite structure of cubic phase above Curie temperature (*T*_c_), which shows the paraelectric phase with no piezoelectricity due to the presence of inversion symmetry with the cation and the anion centre coinciding with each other. The cubic perovskite structure consists of corner-sharing oxygen octahedra with the B cation in the exact centre and with the A cation in the 12-coordinated cuboctahedral position formed by eight octahedra.^54^ In contrast, its distorted structures lacking inversion symmetry (*e.g.* tetragonal, rhombohedral, *etc.*) present piezoelectricity and potential ferroelectricity.^18^ For example, the shift of B ions towards one of the symmetry-allowed direction, *e.g.*, 6 equivalent 〈100〉_pc_ (the subscript ‘pc’ denotes the pseudocubic index) in a tetragonal distortion, from the body centre induces the break of the centrosymmetric structure (the lack of inversion symmetry) and then provides the prerequisite for piezoelectricity. To be more specific, Fig. 1b shows the tetragonal phase structure of BaTiO_3_ below the *T*_c_.

Table 1 lists the mainstream perovskite piezoelectric compounds with a general molecular formula of ABO_3_ and the molecular ferroelectrics with a general chemical formula of ABO_3_ and the organic–inorganic hybrid ferroelectrics with a general chemical formula of ABX_3_ (X means halogen elements). To clearly summarize our discussion below, we intentionally group the ABO_3_ into three categories: A^I^B^V^O_3_, A^II^B^IV^O_3_ and A^III^B^III^O_3_. The superscript roman numerals stand for the valence state of corresponding cations. The construction of solid solutions among these three (*e.g.* BiFeO_3_–BaTiO_3_, BiScO_3_–PbTiO_3_, (Na,Bi)TiO_3_–BaTiO_3_, (Bi,Na)TiO_3_-(Bi,K)TiO_3_–BaTiO_3_, *etc.*) are also possible with the mixed valence states on both A and B sites.^54–60^ As well demonstrated in the previous studies, the construction of solid solutions is a universal and effective approach to high-performance dielectric, piezoelectric, ferroelectric and/or relaxor properties with the modification of phase types, defect chemistry or microstructrues.^61–63^ For example, Lee *et al.* reported a bismuth ferrite and barium titanate solid solution compound, which presents good piezoelectric properties (*d*_33_ = 402 pC N^−1^) and a high Curie temperature (*T*_c_ = 454 °C).^64^ The fundamental origin of improved piezoelectric response basically lies in the flattened free energy landscape, causing the ferroelectric polarization to rotate more easily.

**Table 1:** Typical examples of perovskite ferroelectrics

Structure	Compositions
A^I^B^V^O_3_	LiNbO_3_, KNbO_3_, NaNbO_3_, (K,Na)NbO_3_, *etc.*
A^II^B^IV^O_3_	BaTiO_3_, PbZrO_3_, PbTiO_3_, (Na,Bi)TiO_3_, Pb(Zr,Ti)O_3_, ZnSnO_3_, *etc.*
A^III^B^III^O_3_	BiFeO_3_, BiScO_3_, *etc.*
ABX_3_	Me_3_NCH_2_ClMnCl_3_, Me_3_NCH_2_ClCdCl_3_, *etc.*

##### (i) A^I^B^V^O_3_

**Fig. 2:**
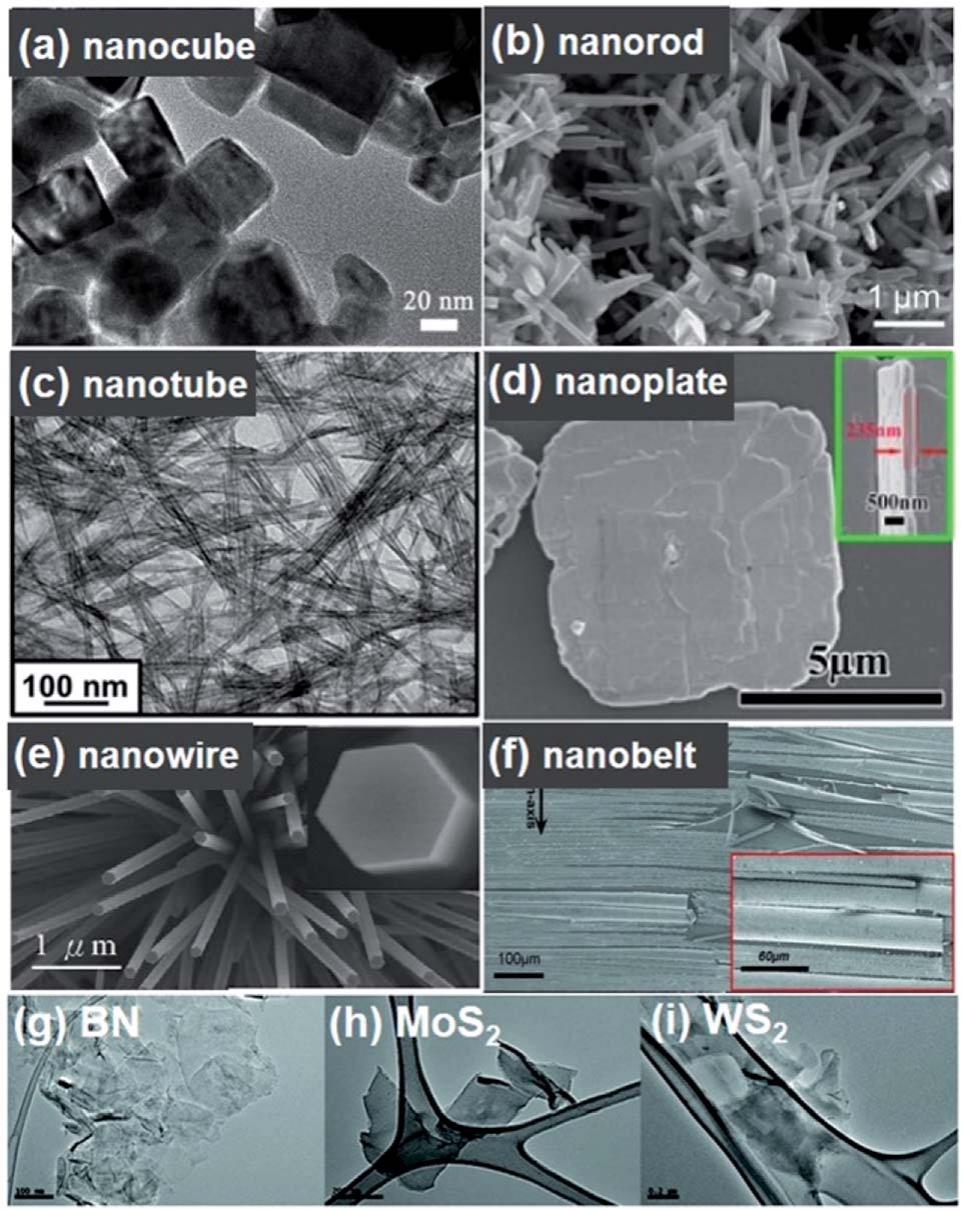
All figures are scanning electron microscopy (SEM) images. (a) (K_0.5_Na_0.5_)NbO_3_ nanocube synthesized by a combustion process using polyacrylic acid (PAA) as a fuel. (b) (K_0.99_Na_0.01_)NbO_3_ nanorods fabricated by a hydrothermal method. (c) BaTiO_3_ nanotubes obtained by a hydrothermal approach. (d) BiFeO_3_ nanoplates synthesized by a hydrothermal approach. (e) Grown ZnO nanowires. (f) GaN nanobelts. (g–i) mechanically exfoliated monolayer of 2D BN, MoS_2_ and WS_2_, respectively. This figure has been adapted/reproduced from ref. 18, 70, 82 and 95–98 with permission from Elsevier, copyright 2016; Elsevier, copyright 2015; American Chemical Society, copyright 2012; American Chemical Society, copyright 2018; Royal Chemical Society, copyright 2020; IOP Science, copyright 2008; and John Wiley & Sons, copyright 2012, respectively.

As a typical A^I^B^V^O_3_ perovskite ferroelectrics, (K_0.5_Na_0.5_)NbO_3_ (KNN) system is characterized by a high Curie temperature (*T*_c_ = 420 °C), good ferroelectric properties (*P*_r_ = 33 μC cm^−2^), and large electromechanical coupling factors, which is powerfully considered as one of the most promising lead-free piezoelectric ceramic materials and suitable for environmental and biological compatibility.^65–68^ Pristine KNN undergoes phase transitions from a paraelectric phase (cubic) to ferroelectric phases (tetragonal, orthorhombic and rhombohedral), and typically presents an orthorhombic symmetry at room temperature.^69^ Among various lead-free piezoelectric ceramic compounds, the KNN system have attracted most attention from the research community not only to macroscale bulk materials but also to nanoscale materials. Nanostructures with various morphologies, (*e.g.* nanoparticles, nanocubes, nanowires, nanofibers, nanoplates, nanofilms, *etc.*) have been demonstrated by various physical and wet chemical methods.^70–72^ 0D (nanocubes) and 1D (nanorods) KNN nanostructures are shown in Fig. 2a and b.^70,73^ Note that more detailed fabrication procedures and morphology modulation will be discussed in Section 2.2.

##### (ii) A^II^B^IV^O_3_

In 1940's, the piezoelectricity of artificially synthesized polycrystalline materials was firstly demonstrated in BaTiO_3_ ferroelectric ceramics. Although it is mainly used as a dielectric material for multi-layered ceramic capacitors (MLCC) nowadays due to its excellent dielectric properties, more and more studies using BaTiO_3_ as a model material to investigate the nanoscale effect of ferroelectrics and piezoelectrics.^18,74^ Pristine BaTiO_3_ undergoes a series of first-order phase transitions as follows: cubic (*Pm*3̄m) to tetragonal (*P*4*mm*), to orthorhombic (*Amm*2), and then to rhombohedral (*R*3*m*) during cooling from high temperatures.^59,75^ As well studied, the construction of morphotropic phase boundary (MPB) or polymorphic phase boundary (PPB) at room temperature was considered as an efficient strategy for higher piezoelectric responses of BaTiO_3_-based ceramics. In 2009, the potential of BaTiO_3_-based materials was renewed for high-performance piezoelectric applications as reporting an extremely high piezoelectric coefficient (*d*_33_ ∼ 620 pC N^−1^) at a composition in Ba(Ti_0.8_Zr_0.2_)O_3_–(Ba_0.7_Ca_0.3_)TiO_3_.^76^ Note that the piezoelectricity of nanomaterials may be different from their bulk counterparts due to the existence of surface and size effect.^77^ Nevertheless, syntheses and uses of nanomaterials largely and inevitably follow the trend of bulk ceramics. Thus, BaTiO_3_-based nanomaterials, especially Ba(Ti_0.8_Zr_0.2_)O_3_–(Ba_0.7_Ca_0.3_)TiO_3_, with various morphologies have been examined by sol–gel process,^78^ supercritical fluids technology,^79^ solvothermal synthesis,^80^ molten salt approach,^81^ and extensively used for lead-free energy and sensor devices since 2009. More details of fabrication and morphology modulation approaches will also be discussed in the subsequent section. Fig. 2c shows the morphology of hydrothermally synthesized BaTiO_3_ nanotubes with 11.8 (±2.3) nm in diameter and 4.1 (±1.2) μm in length.^82^

Even though these types of perovskite piezoelectric ceramics are the simplest composition and the most-friendly material to common engineers, the controls of yield and morphology of 1D type (*i.e.* nanowires) were considered as highly difficult problems.^83^ In recent years, however, the scalable synthesis as well as the ultra-long morphology have been developed through the modulated chemistry.^84–87^

(Na_1/2_Bi_1/2_)TiO_3_ (NBT or BNT), another promising candidate for lead-free piezoelectric materials, was firstly released in 1960.^88,89^ It possesses a perovskite structure at room temperature with a sequence of phase transitions from cubic to tetragonal to rhombohedral at temperatures of ∼540 °C and ∼300 °C, respectively.^90^ NBT system shows strong ferroelectric properties with a large remnant polarization (*P*_r_ = 38 μC cm^−2^) and a high Curie temperature (*T*_c_ = 320 °C). Moreover, there are notable lead-free relaxor ferroelectric ceramics in the NBT-based compositions for high-performance lead-free piezoelectric devices.^91–94^ However, it has been limited for many applications due to its relatively large leakage current and low depolarization temperature (*T*_d_). Nonetheless, there have been many efforts to overcome the disadvantages of NBT systems. In Table 2, we give several typical references related with nanostructures of NBT and its solid solutions with other perovskite systems such as 0.94(Na_1/2_Bi_1/2_)TiO_3_–0.06BaTiO_3_.

**Table 2:** Typical piezoelectric nanomaterials and corresponding fabrication approaches^a^

Approaches	Composition (with references)	Size (nm)	MP*
Solid-state	(K_0.44+*x*_Na_0.52_Li_0.04_)(Nb_0.86_Ta_0.10_Sb_0.04_)O_3_ (ref. 126)	50–200	NP
Solid-state	BaTiO_3_ (ref. 157)	28–105	NP
Solid-state	ZnO (ref. 158)		NP
Solid-state	(K_0.44+*x*_Na_0.52_Li_0.04_)(Nb_0.86_Ta_0.10_Sb_0.04_)O_3_ (ref. 126)	50–200	NP
Solid-state	0.5Ba(Zr_0.2_Ti_0.8_)O_3_-0.5(Ba_0.7_Ca_0.3_)TiO_3_ (ref. 159)	∼500	NP
Sol–gel	0.5Ba(Zr_0.2_Ti_0.8_)O_3_-0.5(Ba_0.7_Ca_0.3_)TiO_3_ (ref. 160)	30–60	NP
Sol–gel	(1−*x*)(K_0.5_Na_0.5_)NbO_3_–*x*LiNbO_3_ (ref. 129)	20–30	NP
Sol–gel	(Na_0.5_Bi_0.5_)TiO3–BaTiO_3_ (ref. 161)		NP
Sol–gel	BiFeO_3_ (ref. 162)		NP
Sol–gel	ZnO (ref. 163)	18–21	NP
Hydrothermal	BaTiO_3_ (ref. 164)		NP
Hydrothermal	Na_0.5_Bi_0.5_TiO_3_ (ref. 165)		NP
Hydrothermal	BiFeO_3_ (ref. 166)		NR
Solvothermal	BaTiO_3_ (ref. 167)	80–100	NP
Molten salt	Na_0.9_K_0.1_NbO_3_ (ref. 168)	∼40	NP
Molten salt	BaTiO_3_ (ref. 169)		
Molten salt	(K_0.5_Na_0.5_)NbO_3_ (ref. 65)		NR
Supercritical fluid	BaTiO_3_ (ref. 79)		
Supercritical fluid	BaTi_1–y_Zr_y_O_3_ (0 ≤ *y* ≤ 1) (ref. 142)	10–25	NP
Electrospinning	V–ZnO (ref. 170)		NF
Electrospinning	(Na_0.5_Bi_0.5_)_0.94_TiO_3_–Ba_0.06_TiO_3_ (ref. 171)		NF
Electrospinning	BaTiO_3_ (ref. 172)		NF
ME**	BN (ref. 173)		2D
ME**	MoS_2_ (ref. 173)		2D
CE***	BN (ref. 174)		2D
CE***	MoS_2_ (ref. 175)		2D

a Note: MP*, ME**, CE*** are the abbreviations of morphology, mechanical exfoliating, and chemical exfoliating process, respectively. The blank means various or vague feature at each research.

##### (iii) A^III^B^III^O_3_

As a well-known multiferroic ceramic material, BiFeO_3_ (BFO) is also considered a candidate for lead-free piezoelectric materials, particularly for high-temperature application due to its very high *T*_c_ (∼830 °C).^99,100^ It exhibits a rhombohedral phase with eight polarization variants at room temperature. Construction of rhombohedral-tetragonal phase boundary could be achieved by alloying other ABO_3_ compounds such as BaTiO_3_, PbTiO_3_, *etc.*, which, then, improves the piezoelectric properties.^101–103^ BFO is the representative one being simultaneously magnetic and strongly ferroelectric (*i.e.* multiferroic) at room temperature, which endows it with an indispensable system for multifunctional devices in specific fields like spintronics.^104^Fig. 2d shows hydrothermally synthesized BiFeO_3_ nanoplates with the thickness around 80 nm.^95^ BFO nanomaterials with other morphologies and related fabrication approaches will be also further discussed in Section 2.2. BFO is also considered as powerful material to modify the ferroelectric properties of BaTiO_3_–SrTiO_3_ solid solution system. The proper ternary system can realize the coexistence of rhombohedral and tetragonal nanoscale domains within a cubic-lattice matrix.^105^ This approach accomplished ultrahigh-energy density lead-free relaxor films.

##### (iv) ABX_3_

Newly-focused perovskite materials, termed as hybrid organic–inorganic or molecular perovskites, have been extensively investigated due to the development of high-performance photovoltaic devices. It also has the piezoelectric and ferroelectric activity, similar with perovskite oxides.^106,107^ For example, the *d*_33_ coefficient of trimethylchloromethyl ammonium trichloromanganese(ii) [Me_3_NCH_2_ClMnCl_3_, (TMCM–MnCl_3_)] was reported as up to ∼185 pC N^−1^, which is comparable to that of BaTiO_3_ (∼190 pC N^−1^).^106^ The advantages of these kinds of perovskite materials lie on low-temperature fabrication, intrinsic flexibility, eco-friendly property, *etc.* However, the piezoelectric properties of this material systems are usually not high, compared to the perovskite oxides. Therefore, the molecular perovskite material is not mainly included in this review because it is not the core of piezoelectric and ferroelectric materials as well as it is important almost only for photovoltaic devices and solar cells as semiconducting materials.

#### 2.1.2 Wurtzite structure

Another essential category in piezoelectric materials is the wurtzite structure such as. ZnO, GaN, InN, ZnS, and so forth where the piezoelectricity also originates from the lack of inversion symmetry in the crystal structure.^108^ They are inherently lead-free and have highly simple stoichiometry, compared to the perovskite materials. Taking ZnO as an example, the stereoscopic illustration of wurtzite structure is shown in Fig. 1b.^53,109^ The tetrahedrally coordinated Zn^2+^ and O^2−^ are stacked layer-by-layer along the *c* axis based on a hexagonal lattice. The charge centre can be separated by external mechanical stress resulting in a structural deformation, which comes out as a piezoelectric potential. Although the composition is very simple and practical, the piezoelectricity of bulk wurtzite materials is much less useful than perovskite ceramics, since the wurtzite structure is not ferroelectric material. This indicates that there is no domain behaviour, only random dipoles in a polycrystalline configuration, *i.e.*, the spontaneous polarization and poling process is impossible. Additionally, the piezoelectric coefficients of these types of materials are much low, compared with the perovskite oxides. However, the piezoelectricity of wurtzite nanomaterials has drawn considerable attentions, because the wurtzite nanomaterials can be synthesized as single crystals which means highly arranged dipole moments dispensing with poling processes or domain controls. More intriguingly, a synergy of piezoelectricity and semiconducting properties, which is called as the piezotronic effect, has also attracted tremendous attentions for multifunctional future electronics.^98,110^ The first prototype piezoelectric nanogenerator (mechanical energy harvester including nanomaterials) was also based on the vertically grown ZnO nanowire forest aligned toward *c* axis.^22^ The ZnO nanowires and GaN nanobelts are presented in Fig. 2e and f, respectively. AlN is another representative wurtzite-structured piezoelectric material. It has been importantly studied for micro-electromechanical systems (MEMS) and biomedical devices because AlN has high biocompatibility and chemical resistivity as well as facile thin film processability with the feature of well crystalline texturing and low-temperature fabrication.^111–113^ Relatively, AlN syntheses of nanopowder and nanostructures have not been studied deeply in fields of piezoelectric properties and applications. Because the syntheses and fabrications of wurtzite materials are relatively easy and very broadly known, it is not the main topic in this review.

#### 2.1.3 Two-dimensional (2D) materials

**Fig. 3:**
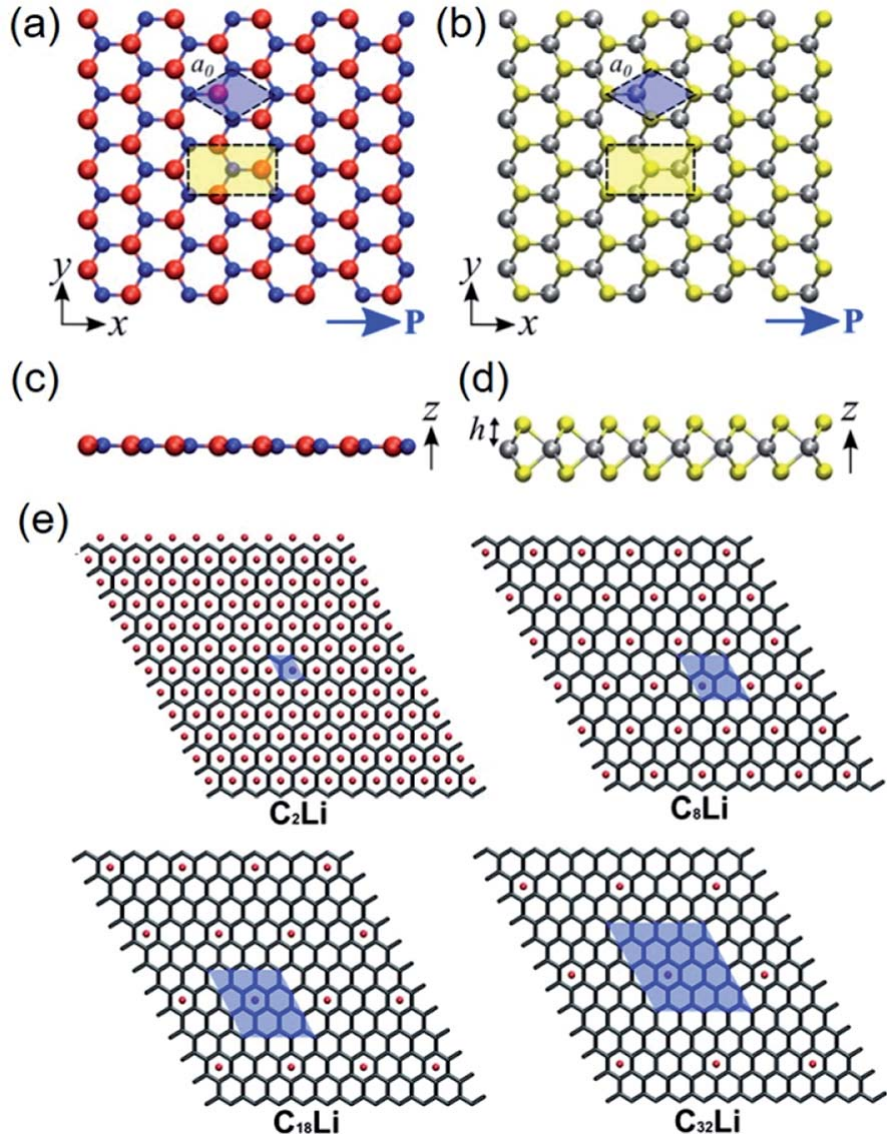
Top-view geometry of monolayer (a) h-BN and (b) trigonal prismatic molybdenum disulfide (2H–MoS_2_) (B atoms: red, N atoms: blue, Mo (transition metal) atoms: silver, S (chalcogenide) atoms: yellow). The axes and directions of piezoelectric polarization are designated. The hexagonal primitive cell is marked as blue regions. The orthorhombic unit cell applied in the density functional theory (DFT) simulations is marked as yellow regions. (c and d) Side-view geometry of the atomically thin h-BN monolayer and the 2H–MoS_2_ monolayer, respectively. (e) Modelling for different concentrations of Li components doped on graphene layers. Unit cell is marked as blue regions, and expressed by the formula unit C_*n*_Li in which *n* = 2, 8, 18, and 32. This figure has been adapted/reproduced from ref. 115 and 125 with permission from American Chemical Society, copyright 2012.

Three general types of piezoelectric 2D nanomaterials, *i.e.*, h-BN, two-dimensional monolayer transition metal dichalcogenides (TMDCs), and modified graphene, are also discussed in the following parts. In 2002, Mele *et al.* reported the macroscopic electric polarization in BN nanotubes.^114^ In addition, the intrinsic piezoelectricity in TMDC nanoscale materials is demonstrated by the density functional theory (DFT) in 2012, as shown in Fig. 3.^115^ The principle of the piezoelectricity of 2D materials are based on the non-centrosymmetry and the lack of inversion symmetry of the 2D crystallographic sheets along the in-plane direction (Fig. 3a–d). Although graphene cannot show intrinsic piezoelectricity, the doped graphene structure can cause piezoelectric effects in accordance with the broken symmetry and local inhomogeneity (Fig. 3e). Mechanical/chemical exfoliation techniques, chemical vapour deposition (CVD), and even photonic approaches were used for the fabrication of 2D materials.^51,116–118^Fig. 2g–i shows the morphology of mechanically exfoliated monolayer BN, MoS_2_, and WS_2_, respectively.^96^ The monolayer of h-BN structure is atomically thin, whereas that of TMDCs consists of two hexagonal planes separated by a plane of metal atoms, as presented by Fig. 2e and d.^19,115^ In recent years, there have been some reports about out-of-plane quasi-piezoelectric properties of 2D materials, resulting from flexoelectric effects by morphological corrugations.^41,119,120^

### 2.2 Fabrication approaches and morphology modulation

A variety of fabrication approaches (*e.g.* hydrothermal method, sol–gel approach, solid–state reaction, electrospinning, molten salt reaction, mechanical exfoliating process, chemical exfoliating process, *etc.*) have been proposed for piezoelectric nanocrystals.^54,121–124^Table 2 lists some typical piezoelectric nanomaterials and corresponding fabrication approaches. The modulation of reaction parameters varies depending on typical fabrication approaches. Surely, the morphology of nanomaterials could be adjusted by reaction time, temperature, raw materials, pH value, and template types. The statements below explain the synthesis and fabrication methods for piezoelectric nanoparticles including 1D, 2D nanomaterials as well as 0D nanoparticles. We explain the representative fabrication methods in this section. It should be noted that some minor or scarce methods are skipped here such as laser ablation.

#### 2.2.1 Solid-state reaction

This is a universal and low-cost physical technique for preparing ferroelectric oxides with a controlled particle size. In most cases, solid–state reaction includes the ball milling or other mechanical pulverization processes, in which the nanoparticle size could be adjusted by the milling parameters such as milling time and speed. The processing procedure for solid-state reaction is similar with the fabrication of ceramics without moulding. For example, Rubio-Marcos *et al.* synthesized the orthorhombic phase (K_0.44+*x*_Na_0.52_Li_0.04_)(Nb_0.86_Ta_0.10_Sb_0.04_)O_3_ nanoparticles by a solid-state reaction.^126^ Raw materials of carbonate and oxides are milled individually to obtain an appropriate particle size distribution. Then, the obtained powders are weighed according to the stoichiometric ratio, and mixed by attrition milling, followed by a calcination at an elevated temperature (700 °C) for reaction and crystallization. The obtained particle size is within a range of 60–200 nm. The calcining temperature and the attrition milling process could be further modulated to adjust the particle size and the phase control. This approach is feasible for high quantity and mass production of nanoparticles. Especially, it is compatible with the synthesis of piezoelectric ceramics. However, it is considerably difficult for 1D and 2D nanostructures using the solid–state reaction approaches in general.

#### 2.2.2 Sol–gel approach

**Fig. 4:**
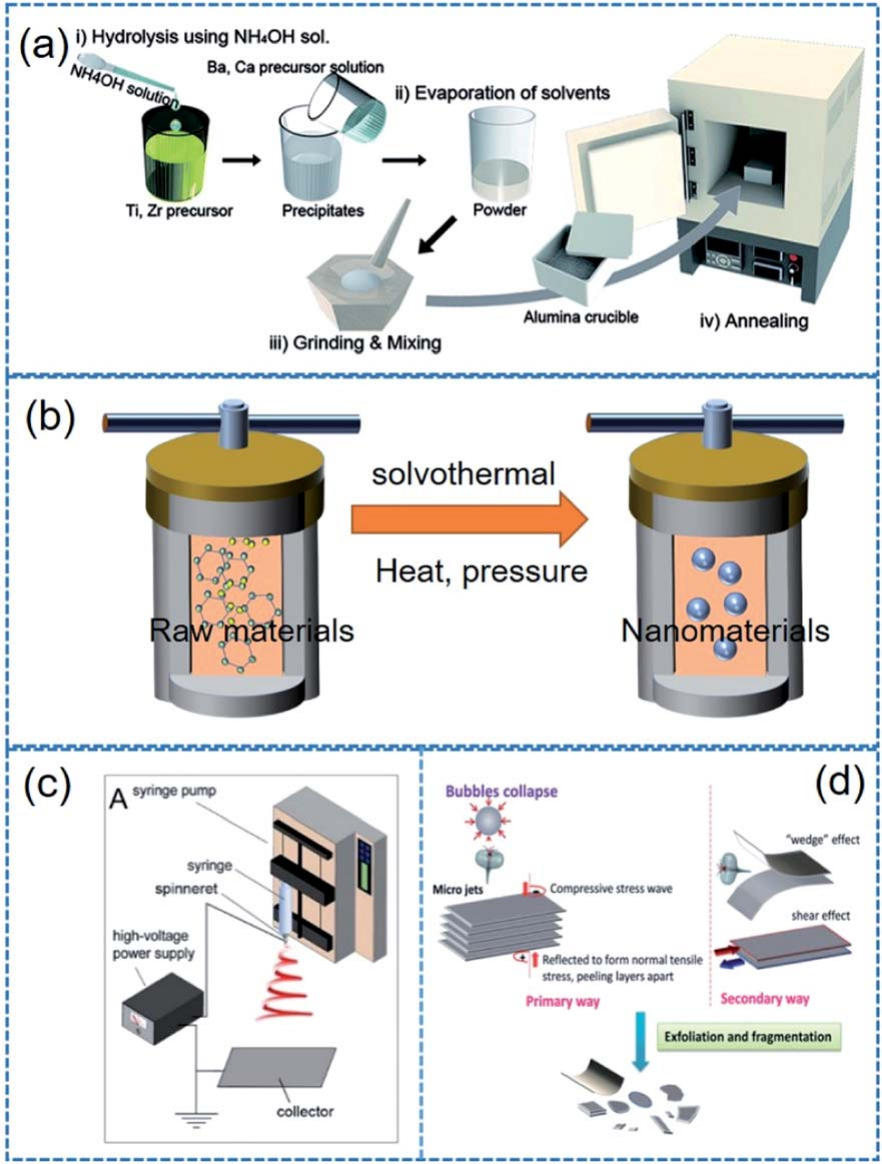
Illustrated flow chart of (a) sol–gel, (b) solvothermal/hydrothermal, (c) electrospinning, (d) mechanical exfoliating process. This figure has been adapted/reproduced from ref. 144, 156 and 159 with permission from American Chemical Society, copyright 2017; Royal Chemical Society, copyright 2015; and Royal Chemical Society, copyright 2016, respectively.

This approach is usually based on the hydrolysis and condensation reactions of molecular precursors such as metal alkoxides and inorganic salts, which are well known as the sol–gel process for film fabrications as well as nanoparticle syntheses. It typically entails the hydrolysis of a solution of precursor molecules to firstly obtain a suspension of colloidal particles (sol), and subsequently the sol is aggregated to from a gel. High-temperature calcination is required for crystallization processes. The advantages of sol–gel process lie on highly homogeneous composition, controllable nanoparticle size, mass production, and compatibility with continuous manufacturing techniques. Therefore, BaTiO_3_, BiFeO_3_, (1 − *x*)(K_0.5_Na_0.5_)NbO_3_–*x*LiNbO_3_, (Na_1/2_Bi_1/2_)TiO_3_–BiFeO_3_, ZnO, *etc.* have been prepared by this approach.^127–131^Fig. 4a shows the basic flow chart of sol–gel process. The microstructure of final products can be varied by solvent evaporation rate, heat treatment condition, temperature, solvent types, and so on. In 2006, for example, Brutchey *et al.* reported a vapour-diffusion sol–gel route to well-defined crystalline BaTiO_3_ nanoparticles at an extremely low temperature (16 °C) in the absence of structure-directing templates.^132^ After that, the vapour-diffusion sol–gel method was largely adopted for fabricating sub-15 nm BaZr_*x*_Ti_1−*x*_O_3_ (0 < *x* < 1) nanocrystals at room temperature.^133^ Note that the particle size of sol–gel derived nanoparticles could be further adjusted by manually grinding and/or mechanical ball milling process. Sol–gel process is relatively costly, and involves complex organic chemistry incompatible sometimes to traditional ceramists.

#### 2.2.3 Hydrothermal or solvothermal synthesis

The solvothermal/hydrothermal technology is widely used to fabricate piezoelectric nanoparticles with diverse morphologies. It should be noted that the hydrothermal technology is based on aqueous solution in the water as a dispersion medium, while solvothermal technology is based on a non-aqueous dispersion medium. They have garnered a considerable attention because they can lead to high-quality crystal powders at low temperatures by one-step reactions. In most cases, raw materials are firstly dissolved and dispersed in a typical solvent and subsequently encapsulated in an autoclave reactor (Fig. 4b). A subsequent heat treatment with a consequent heat-induced high pressure (*i.e.* the resulting reaction temperature becomes higher than the normal boiling point of solvent at ambient pressure) provides the prerequisite for crystallization process.^134^ The nanoscopic morphology could be easily modified by adjusting reaction conditions such as solvents, precursors, temperature, pH, capping reagents/surfactants, *etc.*^20,135,136^ For example, special bowl-like BT nanoparticles (diameter of 100–200 nm) were synthesized by a hydrothermal reaction using Ba(OH)_2_·8H_2_O and TiO_2_ at 180 °C for 72 h.^137^ Demerits of this method include the use of expensive autoclave chambers and the difficulty in monitoring crystal growth during the process.

#### 2.2.4 Molten salt reaction

The molten salt approach is featured by their large quantity yield and environmentally friendly characteristics.^20,138^ During the reaction, the molten salt acts as a reaction medium in which reactants dissolve and precipitate to form the nanoscale crystals. In addition, the morphology and the overall shape of obtained nanomaterials are determined by surface and interfacial energy balance between the molten salt and the constituents.^139^ There are some examples of lead-free piezoelectric nanoparticles synthesized by one of these approaches, as shown in Table 2. For instance, the composition of (K,Na)NbO_3_ nanorods was very well controlled with a specific morphology by a molten salt reaction.^65^ Therefore, the piezoelectric properties of the (K,Na)NbO_3_ nanorods can be more optimized with a clear single-crystalline configuration, which was considered as a difficult problem. In this approach, the separation of final products is highly important. At the last stage, not only the solubility of products but also the interaction between salts and products are critical factors, because sometimes intercalates or solid solutions can result from undesired interactions. This is the major potential disadvantage of molten salt reactions.

#### 2.2.5 Supercritical fluid technology

The supercritical fluid technology is also widely used to form nanoparticles from solutions. It utilizes the precipitation behaviour of solutions around their critical temperature and pressure. There are several sub-classifications in the field of supercritical fluid technology such as static supercritical fluid process, rapid expansion of supercritical solutions (RESS), gas antisolvent process (GAS), precipitation from compressed antisolvent (PCA), and so on.^140^ Because this method has broadly used to synthesize particles for drug delivery, there have been many developed methods using this principle.^141^ In this review, we only deal with RESS due to the limited space for this paper. The RESS consists of the saturation of supercritical fluid (*e.g.* supercritical CO_2_) with solutes, and then the depressurization of this supercritical solution produces a rapid nucleation of the desired materials in the form of nanoparticles which can be collected from the gaseous stream. Supercritical fluid technology is sometimes combined with a hydrothermal synthesis, called the supercritical hydrothermal synthesis. As an example of the supercritical fluid technology, Philippot *et al.* reported very small Ba(Zr,Ti)O_3_ nanoparticles using supercritical fluid conditions with various compositions between Zr and Ti.^142^ Even though the supercritical fluid technology results in a high crystallinity and a homogeneously controlled composition, there are still some problems such as relatively large-sized formation and coagulation.^143^ Therefore, researchers have focused on more advanced processes for small-sized nanoparticles, well-controlled morphology, and high dispersity. There are also some examples of lead-free perovskite oxide nanoparticles fabricated by this approach.

#### 2.2.6 Other viewpoints

##### (i) Electrospinning

Note that the electrospinning is not a synthesis method of nanoparticles. Electrospinning process is more suitable for long 1D microstructures or (sub-)nanostructures. However, we mentioned it here owing to its particular role in the application of piezoelectric nanoparticles. Electrospinning is a simple and versatile technique that relies on the electrostatic force between surface charges to continuously draw nanofibers from a viscoelastic fluid such as particle dispersions or sol–gel solutions.^144^ It has been adopted to successfully fabricate many piezoelectric nanofibers, including (Na_1/2_Bi_1/2_)TiO_3_–BaTiO_3_, BaTiO_3_, 0.5Ba(Zr_0.2_Ti_0.8_)O_3_–0.5(Ba_0.7_Ca_0.3_)TiO_3_, (Na_1/2_Bi_1/2_)TiO_3_, (K_0.5_Na_0.5_)NbO_3_, BiFeO_3_, ZnO, *etc.*^71,145–149^ The basic flow chart of electrospinning is presented in Fig. 4c. For only ceramic fibre structure, a high-temperature calcination and a crystallization must be needed. In contrast, the electrospinning provides composite fibres composed of polymer matrix and incorporated piezoelectric nanoparticles to form organic–inorganic hybrid piezoelectric fibers.^150,151^

##### (ii) Obtaining 2D materials

In 2004, Novoselov used adhesive tapes to exfoliate graphene from graphite, and then initiated the world-wide exploration on 2D materials.^152^ Mechanical exfoliating technology, which is characterized by simplicity and low cost, springs up due to the research fever on 2D materials.^153^ Although the adhesive tape-based mechanical exfoliation is the first leading technique for 2D materials, it is not suitable for making 2D material flake dispersions. Note that it is also highly different from 2D material film fabrications. Therefore, some modified mechanical exfoliation and chemical exfoliation processes were developed to obtain 2D material dispersions such as ball milling, sonication or intercalation (Fig. 4d).^154–156^ In Table 2, we show some examples of 2D materials which were obtained from mechanical or chemical exfoliating process.

## 3. Devices and applications

**Fig. 5:**
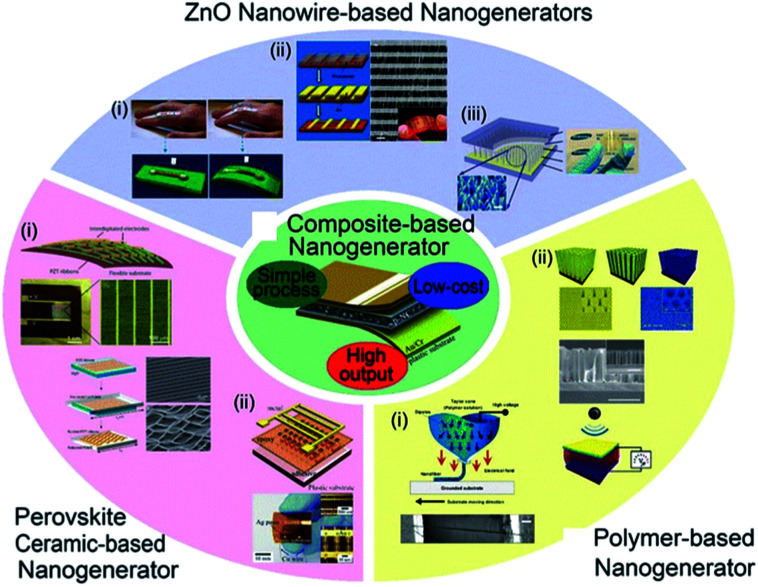
Diverse types of piezoelectric energy harvesters and nanogenerators. This figure has been adapted/reproduced from ref. 196 with permission from Springer, copyright 2016.

The increasing popularity of portable electronics and Internet of things (IoT) demands durable and self-maintaining energy sources, intriguing the extensive research on energy conversion devices such as thermoelectric, photovoltaic, piezoelectric devices, and so forth.^23,155,176–183^ According to other mechanical energy harvesting devices, piezoelectric devices relatively did not have critical drawbacks such as weak humidity resistance and related encapsulation difficulty as well as significant mechanical abrasion problem.^23,184–186^ Among them, piezoelectric energy harvesters, which directly convert mechanical energy input into electrical energy output, have been of an intense interest during the last decade for self-powered and ubiquitous applications.^187–190^ They are highly suitable for indoor and/or isolated environments such as bridge girders, bio-implantation, and broad sensor network systems.^191–193^ Note that the energy harvesters including nanomaterials are often called nanogenerators. Fig. 5 shows various piezoelectric nanogenerators (PNGs) that have been developed till now. Among the diverse piezoelectric nanogenerators, piezocomposite nanogenerators, which were firstly demonstrated by Park *et al.* in 2012,^194^ have highly attracted academic and industrial attentions because of their novel characteristics such as economy, scalability, efficient performance, and mechanically flexible properties.^194–197^ They consist of piezoelectric nanoscale filler and polymer matrix, often with additional mechanical agents.

Two kinds of polymer matrices have been mainly used for piezocomposite nanogenerators, *i.e.*, non-piezoelectric polydimethylsiloxane (PDMS) polymer or poly(vinylidene fluoride) (PVDF)-based piezoelectric polymers. For advanced developments, some other polymer matrices have also been adopted such as Ecoflex silicone rubber,^195^ polyvinyl chloride (PVC),^198^ polycarbonate (PC),^199^ silk fibroin,^200^*etc.* For example, the intrinsic elongation rate of Ecoflex silicone rubber is up to ∼900%, which is a good choice for ultra-stretchable composite nanogenerators.^195^ Silk fibroin can be used as the matrix due to its natural biodegradability, which meets the requirement of biological and eco-friendly electronics.^200^ Besides polymer matrices, a myriad of inorganic piezoelectric fillers have been proposed, which serve as the active phases in piezoelectric nanogenerators.^201,202^

The last several years have witnessed the tremendous progress in lead-free piezoelectric nanoparticles, which endows the possibility for further designing high-performance piezoelectric composite energy harvesters. Enhanced energy storage devices (capacitors) are also another important research field of perovskite-structured nanomaterials and thin films.^203–206^ Nonetheless, we will only focus on the field of energy harvesting devices in this review because the energy storage devices are directly related to the dielectric and ferroelectric physics, rather than piezoelectric and electromechanical couplings.^206–211^ Hence, we will discuss piezoelectric energy harvesters based on lead-free piezoelectric nanoparticles and representative composite design strategies in this part.

### 3.1 Stress reinforcing and dispersing effects

As mentioned earlier, piezoelectric composite nanogenerators are generally fabricated by mixing piezoelectric nanoparticle fillers with a polymer matrix by physical stirring process. This process is characterized by low cost, easy controllability, mass-production compatibility, *etc.* For a long time, especially at the beginning period of piezoelectric nanogenerators, this approach has been considered to be the dominating and widely accepted method. However, there exist critical drawbacks, such as inhomogeneous filler distribution and ineffective stress transfer from external forces to internal piezoceramic components, which severely limit the energy harvesting performance from the active composite layers. To alleviate this problem, Park *et al.* proposed an efficient strategy, which is to introduce 1D conducting nanomaterials, *e.g.* copper nanorods, carbon nanotubes (CNTs), reduced graphene oxide, *etc.*^194,212,213^ It also contributes to reducing internal resistance and reactance for fast polarization switching.^194^

**Fig. 6:**
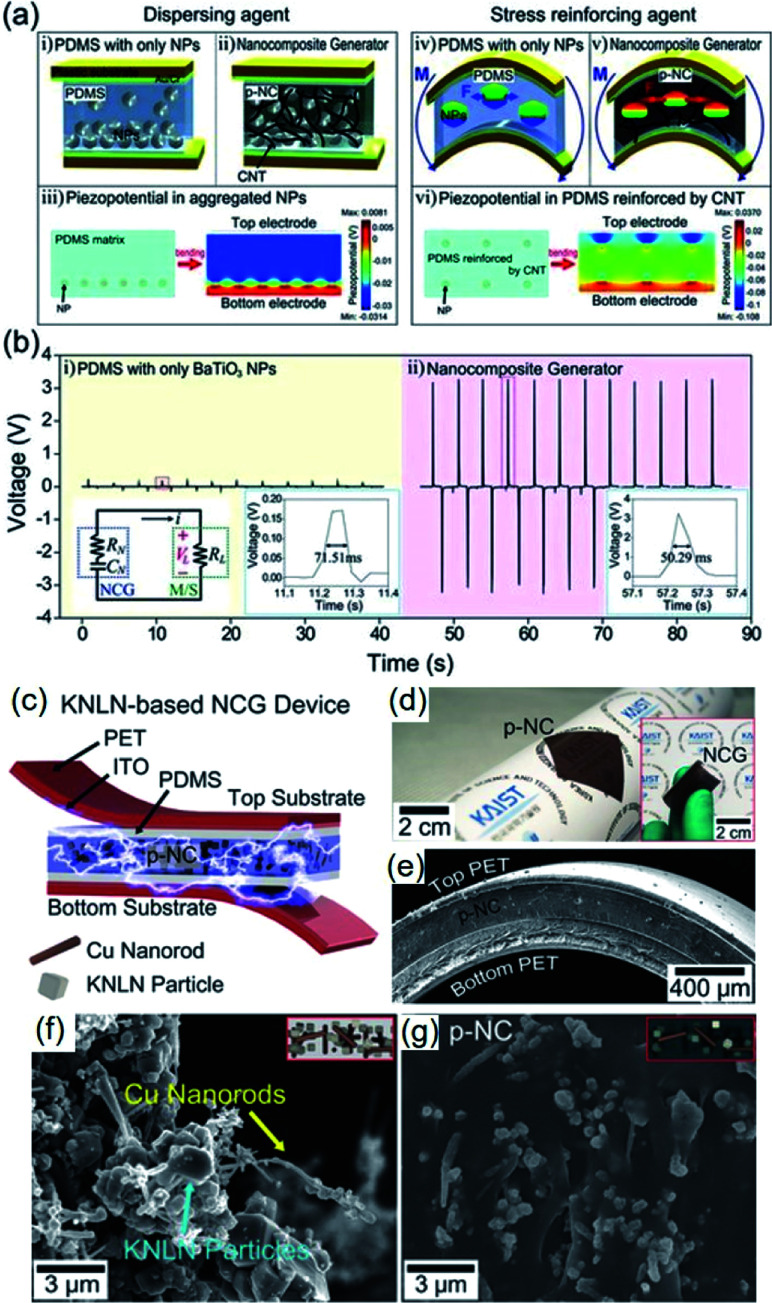
(a) Schematic illustrations of the cross-sectional architecture of nanocomposite generator (abbreviated as NCG) and the simulated piezoelectric potential distributions. The CNTs serve as dispersants (ii) and mechanical reinforcing agents (v) that are well described by the calculated piezoelectric simulations (iii and vi). (b) Voltages generated from the composite devices consisting of only BaTiO_3_ NPs and the NCG structure. The right insets present high resolution of output voltage signals. The left inset is the circuit diagram of the measurement system. (c) Schematic illustration of an NCG structure using high-quality lead-free 0.942(K_0.480_Na_0.535_)NbO_3_–0.058LiNbO_3_ nanoparticles and Cu nanorod fillers with PDMS matrix. (d) Camera photograph of the flexible piezoelectric nanocomposite (p-NC) layer. The inset presents the final NCG device with electrodes/plastic substrates, bent by fingers. (e) Cross-sectional SEM image of the NCG device. (f) Magnified SEM image of the mixed nanomaterials consisting of 0.942(K_0.480_Na_0.535_)NbO_3_–0.058LiNbO_3_ nanoparticles and Cu nanorod before PDMS mixing. (g) SEM image of the cross-sectional p-NC layer with PDMS matrix. This figure has been adapted/reproduced from ref. 194 and 212 with permission from John Wiley & Sons, copyright 2012 and 2014, respectively.

Park *et al.* firstly investigated the positive effects of 1D conductive nanostructures on the mechanical energy harvesting capability of BaTiO_3_/PDMS nanocomposite generators. CNTs serve as physical dispersant, stress reinforcing agent, and conducting functional material, as shown clearly in Fig. 6a. The introduction of CNT results in a dramatically increasing energy harvesting output, as revealed in Fig. 6b. Jeong *et al.* also developed a more high-performance lead-free nanocomposite generator by including metal nanorod fillers (*i.e.* copper nanorod) and high-quality lead-free 0.942(K_0.480_Na_0.535_)NbO_3_–0.058LiNbO_3_ nanoparticles into PDMS matrix.^212^Fig. 6c–e shows the overall device configuration and the real device pictures of the flexible high-output lead-free piezoelectric composite generator. Similar with CNTs, the metal nanorods also acted as an excellent dispersant and a secondary filler in the composite energy harvester, as presented by the scanning electron microscopy (SEM) images (Fig. 6f and g). Note that the stress reinforcement and the dispersion effects are attributed to the 1D geometrical factor of nanotubes, nanorods, and nanowires by effective percolation and entanglement as well as higher Young's modulus within the matrix. Therefore, the 1D piezoelectric (insulating) nanomaterials can also induce self-mechanical improvement. Although the conductive property is not directly affect the mechanical enhancement, it can additionally bestow the ability to present electrical benefits of piezoelectric composites. For example, the conductive filler could reduce the internal resistance of piezoelectric nanocomposite, resulting in the decrement of matching impedance in a final circuit, which is advantageous to device operation. The conductive factor and low internal resistance also shortens a time constant in a RC discharging process, causing the increment of output voltage.^194^ This RC discharging process is also important for poling process of the piezoelectric nanogenerator devices. Recently, AC poling process can be more effective than DC poling process in some cases to achieve not only high piezoelectric performance but also domain direction-related transparency.^214^ This effect may be associated with the RC conductivity in the piezoelectric nanocomposite.

### 3.2 Construction of fibre composite structures

**Fig. 7:**
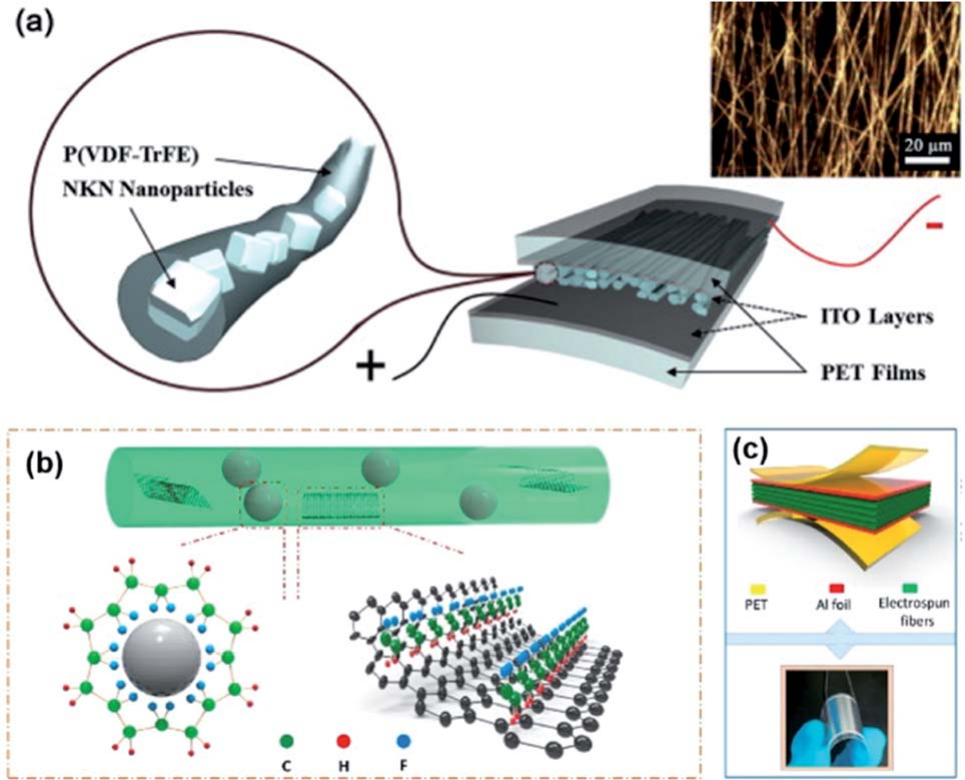
(a) Schematics of a (Na,K)NbO_3_ (NKN)/poly(vinlyidene fluoride–trifluoroethylene) (P(VDF–TrFE)) composite nanofiber-based nanogenerator device (inset: optical microscope image of the piezocomposite nanofibers). (b and c) The mechanism diagram for the formation of β phase of PVDF-based polymer on BT nanoparticles and graphene nanosheets in the nanocomposite fibre and the final device structure with electrodes. This figure has been adapted/reproduced from ref. 70 and 216 with permission from Elsevier, copyright 2015 and 2018, respectively.

Fabrication of core–shell fibre structures is another efficient strategy for high-performance composite nanogenerators for flexible device applications.^70,215–217^Fig. 7 shows the typical examples of this configuration. For these kinds of composite devices, the fabrication procedure could be divided into three steps: (i) mixing piezoelectric nanoparticles and polymer matrix in solvent or dispersant, (ii) spinning methods (mainly electrospinning), (iii) coating electrodes and/or attaching substrate (Fig. 7a). This kind of configuration combines the high flexibility of fibre-structured polymer matrix and the good piezoresponse of embedded piezoelectric nanoparticles. When the matrix is piezoelectric polymer such as PVDF-based copolymers, the piezoelectric nanoparticles affect the dipole of polymeric chain structures sometimes (Fig. 7b).^216^ Due to its intrinsic fibre structure, it is suitable for being integrated with textiles for wearable electronics, as well demonstrated in the previous studies.^218^ Therefore, the use of lead-free piezoelectric nanoparticles is very crucial for biocompatible characteristics of wearable and biomedical device applications. In addition, the integration of electrode materials into each fibre without substrates is still challenging even though it is highly important to achieve real piezoelectric clothes for self-powered wearable electronics. The dielectric properties of polymer matrix in the fibre structure can also affect the device performance. In particular, the electrical breakdown may be one of the representative problems due to the emerging electrical path along with the fibre geometry. We can expect that new approaches to overcome the limitations of polymeric dielectric strength through magnetic or optical processes.^219,220^

### 3.3 Modified distribution of nanoparticles in polymer matrix

**Fig. 8:**
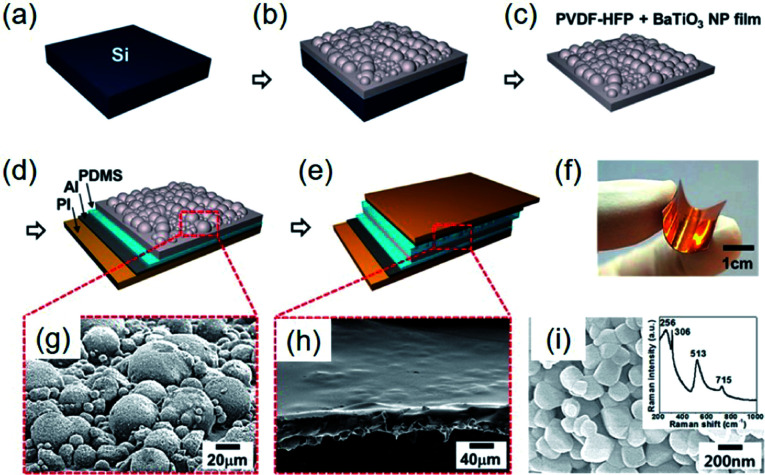
(a–e) Schematic fabrication steps of the hemispherical-structured composite nanogenerator fabrication process. (a) Si wafer as a substrate. (b) BaTiO_3_–P(VDFP–HFP) composite film cured through solvent evaporation after spin coating on the substrate. (c) Peeled off the nanocomposite film from the Si wafer substrate. (d) PDMS-coated Al electrode is attached on the bottom side. (e) PDMS spin coating on BaTiO_3_–P(VDF–HFP) nanocomposite film, followed by the formation of the top electrode. (f) Photograph of the final form of fabricated nanogenerator, showing the mechanical flexibility. (g) SEM image of the BaTiO_3_–P(VDF–HFP) nanocomposite composed of BaTiO_3_–P(VDF–HFP) hemispherical clusters. (h) SEM image of the PDMS-covered BaTiO_3_–P(VDF–HFP) composite layer. (i) SEM image of the BaTiO_3_ nanoparticles used in that research. Inset: a Raman spectrum obtained from the BaTiO_3_ nanoparticles. This figure has been adapted/reproduced from ref. 221 with permission from American Chemical Society, copyright 2014.

In addition to the aforementioned strategies, some special structure designs have been proposed, as exemplarily shown in Fig. 8. The hemispherical BaTiO_3_–poly(vinylidene fluoride-*co*-hexafluoropropylene) (P(VDF–HFP)) clusters were realized by a simple solvent evaporation method, as illustrated in Fig. 8a–c.^221^ After attaching top and bottom electrodes with PDMS buffer layers (Fig. 8d and e), a sandwich-type flexible piezoelectric composite nanogenerator was obtained (Fig. 8f). Using the evaporation of solvent, BaTiO_3_ nanoparticles aggregate together to form hemisphere structures on the composite surface (Fig. 8g). In this research, P(VDF–HFP) served as the tight linker to make aggregated BaTiO_3_ clusters possible. The increasing mechanical energy harvesting performance could actually be ascribed to the effective piezopotential distribution by the hemispherical structures, as also proved by COMSOL Multiphysics simulations.^221^

**Fig. 9:**
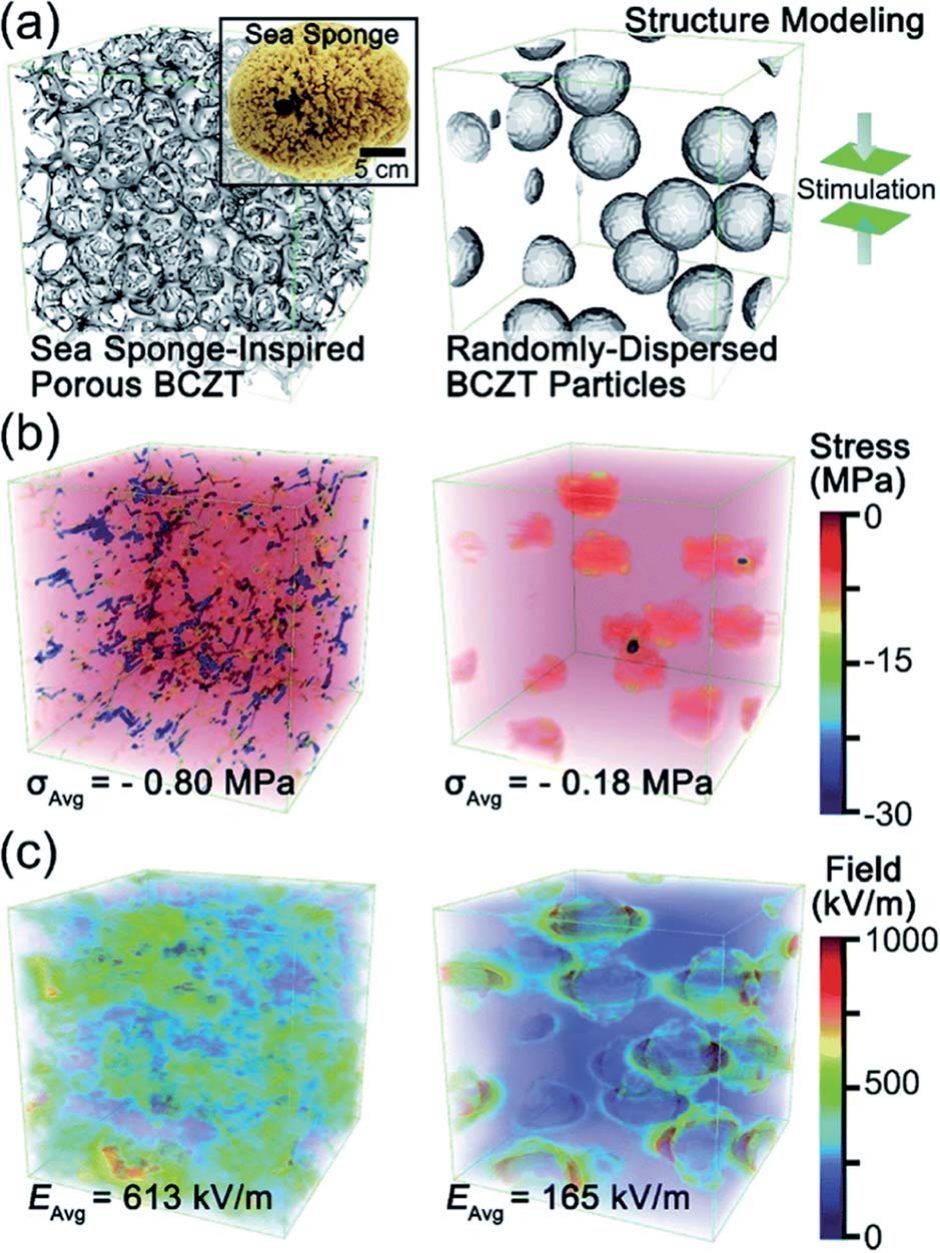
(a) Structural modelling of a sea sponge-inspired BCZT composite and a randomly dispersed BCZT particle-based composite by using the phase-field simulation with the Fourier spectral iterative perturbation method. (b) Computation results of the formed stress within the composites when a compressive strain of 12% is applied. (c) Computation results of the piezopotential-based electrical field generated within the composites when 12% strain is applied. This figure has been adapted/reproduced from ref. 33 with permission from Royal Chemical Society, copyright 2018.

Recently, Zhang *et al.* came up with a novel idea to design a fully three-dimensional (3D) interconnected inorganic filler network with a polymer matrix to dramatically increase the stress transfer ability and demonstrated its beneficial effect on improving piezoelectric energy harvesting capability.^32,33,179^ The structure is presented in Fig. 9. With mimicking the comprehensive properties of sea sponges (*e.g.* mechanical flexibility, recoverability, interconnected network structure, *etc.*), 3D piezoelectric ceramic skeleton was fabricated by combining the lead-free piezoelectric sol–gel processed (Ba,Ca)(Zr,Ti)O_3_ (BCZT) and the suitable calcination/crystallization. The 3D interconnected porous framework structure (the left of Fig. 9a) is based on the natural sea sponge (the inset of Fig. 9a). On the other hand, the right of Fig. 9a is the structure of the conventional polymer composite containing randomly dispersed particles. Regardless of the particle size of piezoelectric ceramic fillers in the polymer matrix, the conventional polymer–ceramic composite has almost the same configuration with the right of Fig. 9a. It should be mentioned at least briefly that the drawback of this kind of structure lies on the problems as follows: (i) the aggregation of fillers due to the randomly dispersed nanoparticles and (ii) the deteriorated stress-transfer ability because the low elastic modulus of polymer matrix. The phase-field simulations clearly demonstrated that the stress distribution in the 3D interconnected structure is much more uniform than that in composites containing randomly distributed particles- under the same applied strain, as shown in Fig. 9b. Moreover, the average stress in the 3D interconnected composite is about 4 times higher than that in the random-particle-based polymer composite. The reinforced mechanical response of the bioinspired structure is apparently due to the fact that the interconnected BCZT 3D structure is capable of efficiently transferring stress from outside to the ceramic fillers, whereas the random-particle-based composite relaxes and dissipates most of the stress within the soft polymer matrix rather than transferring it to the BCZT ceramic particles. The generated piezoelectric potential of 3D interconnected composite is, indeed, greater than that of random-particle-based composite, as presented in Fig. 9c. As expected, the energy harvesting ability of the 3D interconnected composite is superior to the random-particle-based composite. This research guided researchers to a new approach to the application of lead-free piezoelectric nanomaterials. This composite design is also available directly using nanoparticle dispersions without sol–gel solutions using accompanied fibril dispersion.^32^

### 3.4 Incorporation of 2D piezoelectric nanofiller materials in polymer matrix

**Fig. 10:**
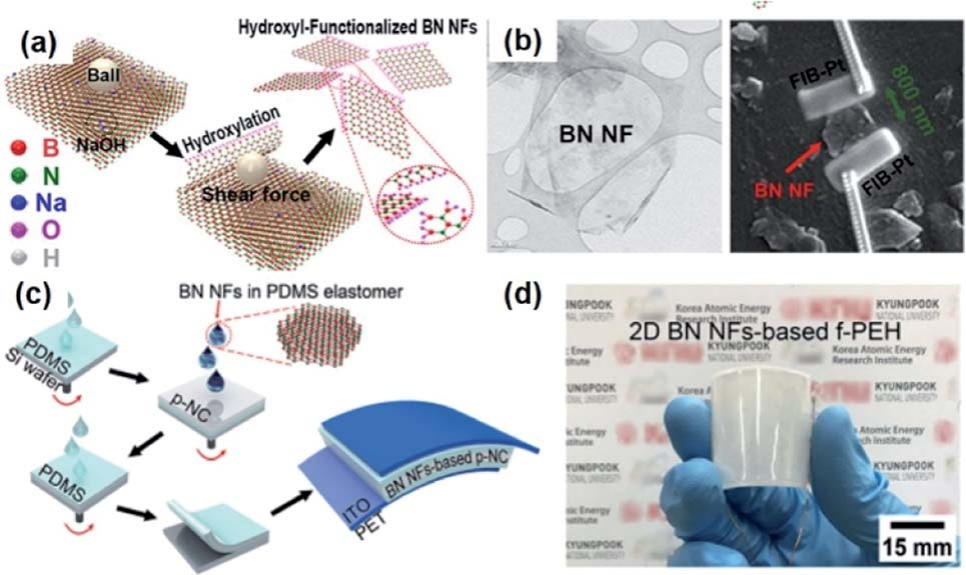
(a) Schematic illustration of the sequential process for fabricating 2D h-BN nanoflakes. (b) Transmission electron microscopy (TEM) image of a h-BN nanoflake and the SEM image of a h-BN single nanoflake connected to two Au line electrodes formed by the focused ion beam (FIB)-assisted deposition method. (c) Illustration of the device fabrication of piezoelectric h-BN nanoflakes-based elastomeric nanocomposite generator and (d) its real photograph presenting flexible configuration. This figure has been adapted/reproduced from ref. 155 with permission from American Chemical Society, copyright 2019.

2D materials in the view of piezoelectricity have been considered a unique category for specific piezoelectric device and applications.^222,223^ Recently, the device configuration for composite nanogenerators has intrigued researchers' attentions.^224^Fig. 10a shows the method for obtaining h-BN nanoflakes using mechanochemcial exfoliation. First, the h-BN nanoflakes were directly transferred to line-patterned electrodes to evaluate the piezoelectric energy harvesting property of single h-BN nanoflake, as presented in Fig. 10b.^155^ The piezoelectric voltage coefficient of a single BN nanoflake and the mechanical energy harvesting performance of BN based nanongenerator were quantified. Finally, the 2D piezoelectric h-BN nanoflakes were embedded into the PDMS elastomer matrix to fabricate a nanocomposite generator showing the energy harvesting performance of ∼9 V and ∼200 nA (Fig. 10c and d). In this configuration, the 2D nanoflakes act as the piezoelectric nanoparticles and nanofillers in the conventional nanocomposite generators. Nevertheless, the 2D piezoelectric material-based composite should be more investigated, because the ferroelectric properties of 2D materials are still veiled in spite of their piezoelectric characteristics. Hence, it is dim whether the external poling is possible or not for randomly distributed 2D nanomaterials. If the 2D material is only piezoelectric but not ferroelectric, the piezoelectric dipole within the piezocomposite must also be random and cannot be aligned in a unified direction (like the composite of ZnO particles), which means the composite configuration of piezoelectric 2D materials is not reliable in contrast to the previously reported plane configuration of 2D sheets.^225^

### 3.5 Applications by energy harvesting signals

As stated above, we summarized several typical strategies that researchers proposed to improve the performance of piezoelectric nanogenerators using lead-free perovskite nanoparticles. The high-output performance as well as the facile fabrication process make the nanogenerators qualified in some intriguing fields such as soft-robotics, wearable sensing systems, cell stimulation and so forth.

#### 3.5.1 Self-powered sensors

Mechanical sensors play vital roles in current industry, and also serve as the hot topic in scientific community for future IoT applications. Beyond conventional ones, the concept of self-powered sensors can be achieved by direct piezoelectric signals from the piezoelectric devices.^226–229^ The main device structure of piezoelectric self-powered sensor is very similar to that of piezoelectric nanogenerators, the piezoelectric signals serve as mechanical detection output, not energy, in the case of self-powered sensor. Although piezoelectric sensors have been used widely in commercial application (*e.g.*, ultrasonic sensor, accelerometer, *etc.*), it should be mentioned that the recently-developed self-powered piezoelectric sensors are related to wearable and IoT electronics for general applications. This important application part has been also demonstrated by the lead-free piezoelectric nanocomposite. Chen *et al.* suggested a high-output flexible piezoelectric nanocomposite generator using nanocomposite micropillar array of P(VDF–TrFE) and BaTiO_3_ nanoparticles for self-powered sensor applications as well as energy harvesting devices.^229^ The lead-free piezoelectric composite device could be applied as self-powered flexible sensor system in a non-contact mode to detect air pressure and as wearable sensor unit to detect human vital signs such as breath and heartbeat pulse. According to the recent process in the machine learning approach, new self-powered piezoelectric sensors have been also developed. Highly-sensitive multi-tunable acoustic sensors were studied by the inspiration of basilar membrane.^230^ It was additionally merged with the machine-learning method to recognize individual speakers. It exhibited noteworthy speaker recognition rate of 97.5% to be comparable with the reference microphone.^231^ These up-to-date technologies can be also applied to piezoelectric nanocomposite devices with performance enhancement.

#### 3.5.2 Piezoelectric bio-interfaces for biological stimulation

Until now, exploiting the unique applications of piezoelectric devices in biomedical fields has got tremendous attentions worldwide.^232,233^ Electrical stimulation for cells and tissues is considered as an important approach to interaction with living matter, which has been traditionally adopted in the clinical practice for a wide range of pathological conditions.^234^ Taking advantage of the generated electrical signals from piezoelectric materials and devices, they could be utilized for electrical stimulation to biological elements. Genchi *et al.* showed the application of P(VDF–TrFE) and BaTiO_3_ nanoparticles lead-free piezoelectric nanocomposite films to stimulate and promote the differentiation of SH-SY5Y neuroblastoma cells.^235^ The researchers determined the emission of significantly longer neurites by the P(VDF–TrFE) and BaTiO_3_ nanoparticles-based composite film due to its enhanced piezoelectric property, compared to the pure P(VDF–TrFE) film. It should be noted that the composite-type piezoelectric materials present benign mechanical properties and piezoelectric responses for the compatibility to biological matter. Surely, the lead-free feature is highly important for biological applications. Biocompatibility of materials is also important issue for biological applications. Although there are many researches to investigate biocompatibility of both lead-based and lead-free piezoelectric ceramic films, evaluating that of nanofillers (*e.g.*, nanoparticles, nanowires, *etc.*) is not the main stream. Nevertheless, we could know that nanofiller materials are biocompatible with lead-free characteristics.^236^

## 4. Summary and outlook

Piezoelectric nanomaterials, in particular environmentally friendly lead-free piezoelectrics, have been an important class of materials both for fundamental research and applications for the past 20 years. It is predicted that piezoelectric properties at nanometer scales would be dramatically different from those of bulk materials or polycrystalline films. Fortunately, the complex interplay between multiple degrees of unprecedented output performances of these nanomaterials demonstrates that techniques developed to investigate them have been applied to other research fields with a great success. Each year brings new insights and new understanding of these complex materials as a new potential for energy harvesting applications. Specifically, as an important type of energy devices, piezo-nanocomposite-based energy harvesters have attracted great attention by the research community due to a broad spectrum of their unique applications such as self-powered portable sensors, transportation, energy distribution, smart home, military weapons, and biomedical devices, and so on.

It clearly indicates that this complex class of materials will continue to attract interest as their performance in the existing devices is improved and novel applications are developed in the years to come. In this review, we provided a synopsis of the major developments and achievements in the current and past years in the area of lead-free piezoelectric nanomaterials and related energy harvesting devices with a strong emphasis on work blazing a new trail in terms of methods and control of material properties at scales, understanding of size effects, and routes to develop new functionalities and improved performance in piezoelectric nanomaterials-based energy harvesting devices.

This review began with a short introduction and a brief discussion of several representative lead-free piezoelectric nanoparticles with various chemical compositions and different crystal structures, followed by the detailed discussion on the state-of-the-art for lead-free piezoelectric nanoparticles with diverse morphological configurations and chemical features, and synthesis methodologies such as solid-state reactions, sol–gel methods, hydrothermal or solvothermal syntheses, molten salt reactions, supercritical fluid technologies, and so on. Then we commented on the applications of lead-free piezoelectric nanoparticles, especially in piezoelectric nanocomposite-based energy harvesting device applications. Finally, we completed this review with a short summary and future outlook of lead-free piezoelectric nanomaterials and devices.

Despite the progress accomplished through those approaches, several issues still remain to be addressed for further improvement. We would like to wrap-up this review with brief comments for a better perspective of this field as follows.

(i) Further clarification of the underlying physics of piezoelectric mechanism at nanoscale.

(ii) Finding new scalable and mass-producible fabrication methods.

(iii) Development of a novel characterization tool or method to evaluate the properties of piezoelectric nanostructures.

(iv) A better understanding of degradation and failure of lead-free piezoelectric nanomaterials and devices using macro- and microscopic measurements and accurate simulation and modelling.

(v) Innovative device designs based on the optimal input parameters of lead-free piezoelectric nanomaterials; thus, guiding and supporting the understanding of performance by comparison of experiment and simulation.

## Conflicts of interest

There are no conflicts to declare.

## Supplementary Material
